# GENDER AND HEALTH IMPLICATIONS OF EXTENDING WORKING LIFE: CROSS-NATIONAL PERSPECTIVES

**DOI:** 10.1093/geroni/igz038.3022

**Published:** 2019-11-08

**Authors:** Aine Ni Leime, Debra A Street

**Affiliations:** 1 National University of Ireland, Galway Ireland, Galway, Ireland, Ireland; 2 State University of New York at Buffalo, Buffalo, New York, United States

## Abstract

This symposium addresses the issue of extended working life policy by considering the influence of gender and health on the experiences of older workers. In response to population ageing, policies designed to extend working life have been introduced in many countries. These policies include raising state pension age and linking the amount of state pensions more closely to years spent in paid employment. Such policies tend to be undifferentiated by gender or health status – in most countries, state pension age has been raised to the same age for men and women. Yet, research evidence indicates that women in all countries are disadvantaged in relation to employment at older ages and pensions. There are also health inequalities for older workers, depending on their occupation and whether they are in precarious or secure employment. Extended working life is of pressing societal concern. This symposium brings together the work of a group of leading international scholars who have been researching and reflecting on its implications in a forthcoming book on the topic across 34 countries. The symposium begins with an overview and analysis of the empirical landscape of older employment and pension policy by Martina Rasticova and Jim Ogg; Paper 2 offers a discussion of the theoretical perspectives and policy debates across 34 countries by Clary Krekula; there will be an analysis of extended working life policy in Ireland by Aine Ni Leime and a final presentation synthesising policy recommendations and mapping future research directions in extended working life by Debra Street.

